# Quantification of biomarker functionality predicts patient outcomes

**DOI:** 10.1038/s41416-021-01291-3

**Published:** 2021-03-15

**Authors:** Banafshé Larijani, James Miles, Stephen G. Ward, Peter J. Parker

**Affiliations:** 1 FASTBASE Solutions S.L., Kabi 612 Scientific and Technology Park of Bizkaia, Derio, 48160 Spain; 2 Cell Biophysics Laboratory, Ikerbasque, Basque Foundation for Science, Research Centre for Experimental Marine Biology and Biotechnology (PiE) & Instituto Biofisikia (UPV/EHU, CSIC), University of the Basque Country, Leioa, Biscay Spain; 3 Cell Biophysics Laboratory, Department of Pharmacy and Pharmacology & Department of Physics, Centre for Therapeutic Innovation, University of Bath, Bath, UK; 4 Early Phase Trials and Sarcoma, Institut Bergonié, Bordeaux, France; 5 Leukocyte Biology Laboratory, Centre for Therapeutic Innovation & Department of Pharmacy and Pharmacology, University of Bath, Bath, UK; 6 Protein Phosphorylation Laboratory, The Francis Crick Institute, London, UK; 7 School of Cancer and Pharmaceutical Sciences, King’s College London, London, UK

**Keywords:** Cancer, Oncology, Lung cancer

## Abstract

Implementation of a quantitative molecular imaging method (iFRET), which determines receptor–ligand interactions, has led to the finding that patients with a low extent of PD-1/PD-L1 interaction in metastatic NSCLC, and malignant melanoma, display significantly worsened overall survival compared to those with a high level of interaction.

## Main

The hallmarks of cancer described by Hanahan and Weinberg illustrate the necessary conditions for the manifestation of malignant neoplastic diseases.^1^ One of these conditions is the evasion of immune-detection and avoidance of immune destruction. A mechanism by which cancers may evade immune-surveillance is dysregulation of inhibitory immune-checkpoints. Immune-checkpoints are comprised of inhibitory receptors, found on the cell surface of immune-cells, and cognate ligands, expressed on antigen presenting cells. The programmed death receptor 1 (PD-1) is an example of an immune-checkpoint receptor. When engaged with is complementary ligand, programmed death receptor ligand 1 (PD-L1), there is a reduction of immune activation. This is in part thought to be facilitated by the recruitment of the tyrosine phosphatases SHP-1 and SHP-2 to the ITSM of PD-1.^2^ Cancers may dysregulate this checkpoint by upregulating PD-L1, thus facilitating an evasion of immune surveillance and destruction by tumour-specific T lymphocytes.

Currently, monoclonal antibodies can be used to block PD-1/PDL-1 interactions, thus restoring immune-mediated tumour detection and destruction. Whilst these therapies have shown promising therapeutic benefit, low response rates are encountered. In a recent study of NSCLC patients, those with a tumour proportion score of 50% or more exhibited increased response to pembrolizumab. However, only a 41% response rate was achieved in this cohort.^3^ To select patients who are likely to benefit from these treatments, a number of immunohistochemistry (IHC)-based assays are used to assess PD-L1 expression, in effect using ligand expression as a surrogate of checkpoint engagement. This indirect determination of checkpoint function has not proved effective in stratifying patients into treatment groups.

**Fig. 1:**
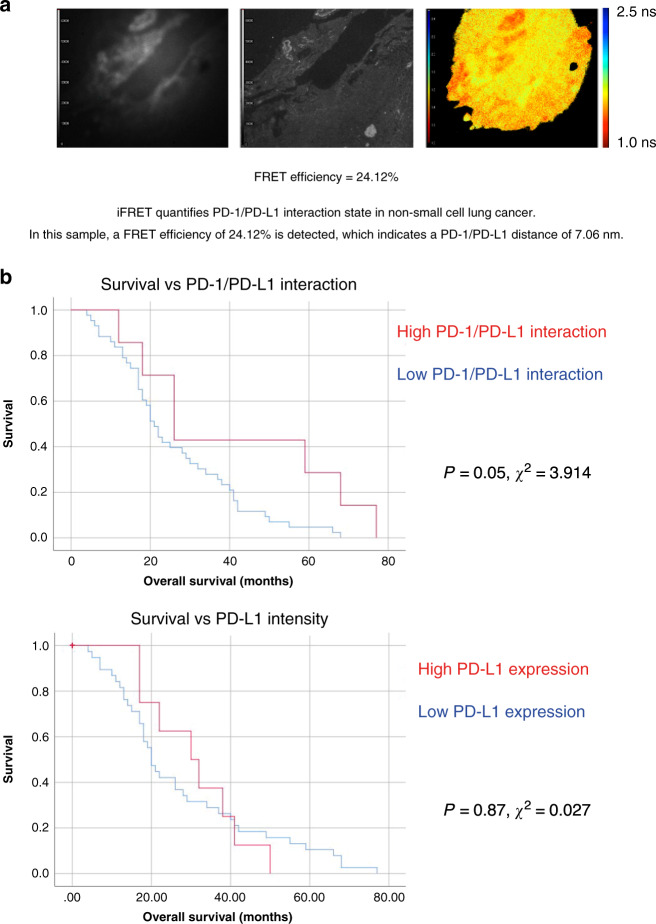
iFRET quantifies PD-1/PD-L1 interaction state with patient outcome in malignant melanoma and NSCLC. **a** iFRET, a two-site assay simultaneously labels PD-1 and PD-L1 in patient samples. This allows the detection and quantification of PD-1 and PD-L1 interaction, alongside receptor and ligand expression. The black and white intensity maps show PD-1 and PD-L1 expression in an FFPE NSCLC sample. The pseudocolour lifetime map indicates FRET occurring between the labelled PD-1 receptor and PD-L1 ligand. This is correlated to a FRET efficiency of 20.77% and a receptor–ligand distance of 7.29 nm. **b** iFRET correlates PD-1/PD-L1 interaction state with overall survival in malignant melanoma. (Top) Kaplan–Meier curve indicates that patients with a higher PD-1/PD-L1 interaction have a significantly worsened overall survival (*p* = 0.05, *χ*^2^ = 3.914). (Bottom) Kaplan–Meier curve correlates overall survival with PD-L1 expression. Here, PD-L1 expression does not correlate with patient outcome (*p* = 0.87, *χ*^2^ = 0.027). Modified from Sanchez-Magraner et al.^5^.

A recent study assessed the efficacy of anti-PD-1/PD-L1 therapies in lung carcinoma, renal carcinoma and melanoma. In this study, patients’ PD-L1 expression was assessed and patients were classified as PD-L1 positive or PD-L1 negative. Here, therapeutic benefit was seen in PD-L1 negative patients, implying that ligand expression is not a suitable biomarker to select patients for immune-checkpoint therapies.^4^ Hence, in order to overcome the shortcomings of IHC-based assays we developed a molecular imaging assay which quantifies PD-1/PD-L1 interaction states, alongside receptor and ligand expression (Fig. 1a).^5^ This assay is termed iFRET. Alternative assays exist which attempt to measure PD-1/PD-L1 interaction, however unlike iFRET these report on distances that likely reflect juxtaposition and cannot be relied upon to accurately report on checkpoint receptor engagement. For example, others have previously used an imaging algorithm which determines when PD-1^+^ and PD-L1^+^ cells are in close proximity (≤20 μm).^6^ However, these distances are greater than the diameter of a cell. By comparison, iFRET reports events at the 1–10 nm scale and can directly measure intercellular protein-protein interactions.

The aforementioned iFRET assay was first compared with the Roche Ventana SP142 assay which is currently used to assess clinical PD-L1 expression. Crucially, the iFRET assay was able to detect a PD-1/PD-L1 interaction in 10 of the 11 PD-L1 negative ccRCC patients.^5^ This demonstrates that ligand expression is a poor surrogate of receptor engagement and that the direct functional PD-1/PD-L1 interaction needs to be quantified. Following this analysis, iFRET was used to assess PD-1/PD-L1 interaction states in 176 malignant melanoma patients with known clinical outcomes. One hundred and fifty-nine of these patients were assessed by IHC methods to determine their PD-L1 expression profiles. Of the 159 patients, 117 were PD-L1 negative and 42 were PD-L1 positive. In the PD-L1 negative group, 58 patients showed checkpoint interaction and 19 of 42 PD-L1 positive group showed no interaction state.^5^ This once again highlights the need for direct assessment of checkpoint interaction, and subsequent functionality, rather than indirect determination through the surrogate of ligand expression. Current methods are creating a scenario wherein patients may be enduring the side-effects of immune-checkpoint inhibition to gain no therapeutic benefit. Conversely, patients may miss these novel treatment opportunities which can potentially deliver increased survival rates.

As patient survival is often correlated to biomarker expression, a potential correlation between PD-1/PD-L1 and overall survival was assessed. We demonstrated firstly in melanoma that PD-L1 expression did not correlate to overall survival (*p* = 0.87) (Fig. 1b). Conversely, patient’s PD-1/PD-L1 interaction state correlated with overall survival (*p* = 0.05). Here, those with a lower PD-1/PD-L1 interaction state experienced a significantly worsened overall survival (Fig. 1b).^5^ Following this, PD-1/PD-L1 interaction states were quantified in a cohort of anti-PD-1 treated metastatic NSCLC patients. As observed in the melanoma cohort, PD-L1 expression did not correlate with overall survival. In this NSCLC post-treatment cohort, those with a lower PD-1/PD-L1 interaction state again had a significantly worsened overall survival (*p* = 0.05).^5^

Current literature suggests that an increase in PD-1/PD-L1 interaction would cause a decrease in overall survival due to immune-suppression. However, the aforementioned results suggest that in the cohorts examined, a higher interaction state may correlate with improved survival. It is surmised that a high PD-1/PD-L1 interaction state infers tumour selection within patients, resulting in a subset of patients whose tumour facilitate immune-evasion via PD-1/PD-L1 interaction. It is this subset of patients who are predicted to respond to anti-PD-1/PD-L1 therapies and possibly should be considered for these treatments. Moreover, it remains unknown what extent of PD-1/PD-L1 disengagement is required to increase immune-mediated tumour destruction by tumour infiltrating lymphocytes.

As the pharmacodynamics of immune-checkpoint interactions are not monitored, it could be that only a partial or transient disengagement of PD-1/PD-L1 is required to observe an improved clinical outcome. It would be beneficial therefore to use iFRET to assess checkpoint interaction over time to observe how these checkpoint interactions evolve after treatment. It could be that the use of anti-PD-1 therapeutic antibodies results in a thresholding of T lymphocytes. In this instance, after treatment with anti-PD-1 therapies, a subset of T lymphocytes undergo reactivation and are polarised towards the tumour via recognition of tumour antigens. The increase of patient survival in those with a high interaction state may highlight that only a partial reactivation of T lymphocytes is needed to re-mount an immune response.

Those patients with a low level of interaction and worsened survival may nevertheless benefit from alternative immune therapies. These tumours may evade the immune system by dysregulating CTLA-4/CD-80 or other immune-checkpoint interactions. Furthermore, no tumour will discretely dysregulate one pathway, in fact, a tumour may evolve to evade host immune response by modulating multiple pathways simultaneously, indicating a patient group who would benefit from dual checkpoint inhibitor therapies.^7,8^ In these instances, the functionality of multiple biomarkers should be quantified simultaneously in order to tailor patients to the most efficacious therapies.

The approach developed can be regarded as a companion diagnostic tool and can be utilised alongside routine IHC PD-L1 analysis using sections from the same fixed biopsies. In a wider context, this approach can be used to determine biomarker functionality to identify novel predictive biomarkers in a range of pathologies.

## Data Availability

The original data are with Cancer Research where the article is published.
